# HiRAND: A novel GCN semi-supervised deep learning-based framework for classification and feature selection in drug research and development

**DOI:** 10.3389/fonc.2023.1047556

**Published:** 2023-01-26

**Authors:** Yue Huang, Zhiwei Rong, Liuchao Zhang, Zhenyi Xu, Jianxin Ji, Jia He, Weisha Liu, Yan Hou, Kang Li

**Affiliations:** ^1^ Department of Biostatistics, School of Public Health, Harbin Medical University, Harbin, China; ^2^ Department of Biostatistics, School of Public Health, Peking University, Beijing, China

**Keywords:** semi-supervised, drug response, deep learning, prediction, graph convolution network

## Abstract

The prediction of response to drugs before initiating therapy based on transcriptome data is a major challenge. However, identifying effective drug response label data costs time and resources. Methods available often predict poorly and fail to identify robust biomarkers due to the curse of dimensionality: high dimensionality and low sample size. Therefore, this necessitates the development of predictive models to effectively predict the response to drugs using limited labeled data while being interpretable. In this study, we report a novel Hierarchical Graph Random Neural Networks (HiRAND) framework to predict the drug response using transcriptome data of few labeled data and additional unlabeled data. HiRAND completes the information integration of the gene graph and sample graph by graph convolutional network (GCN). The innovation of our model is leveraging data augmentation strategy to solve the dilemma of limited labeled data and using consistency regularization to optimize the prediction consistency of unlabeled data across different data augmentations. The results showed that HiRAND achieved better performance than competitive methods in various prediction scenarios, including both simulation data and multiple drug response data. We found that the prediction ability of HiRAND in the drug vorinostat showed the best results across all 62 drugs. In addition, HiRAND was interpreted to identify the key genes most important to vorinostat response, highlighting critical roles for ribosomal protein-related genes in the response to histone deacetylase inhibition. Our HiRAND could be utilized as an efficient framework for improving the drug response prediction performance using few labeled data.

## Introduction

Precision medicine seeks to customize medical treatments based on the genetic characteristics of each patient. Essentially, this is the process of tailoring proactive and preventive care in order to maximize medical efficacy and cost-effectiveness (1). A personalized approach in cancer therapeutics has long been a noble goal for both clinicians and patients. Despite the undeniable success of certain targeted therapeutic approaches (2), unfortunately, they often provide benefits only to a few patients (3), and almost inevitably, patients tend to develop resistance to these targeted therapies as well (4). Tumor subtype and cancer genome evolution, resulting in intratumor heterogeneity, remain the main challenges to successful cancer personalizing treatment (5). Machine learning methodologies, including the emerging deep learning models, are an important component of the analysis to allow for the classification and prediction of outcomes for individuals and populations (6). For example, researchers have developed logistic regression (7) and extreme gradient boosting (XGBoost) (8)-based classifiers that predict drug resistance based on treatment-naive (A person is considered to be “treatment-naive” if he or she has never undergone treatment for a particular illness) genomic, transcriptomic profiles and clinical characteristics. In addition, the deep learning methods, including convolutional neural networks (CNNs) (9–13) and deep neural networks (DNNs) (14–17), are applied to predict the prognosis or response to therapy using gene expression, copy-number alteration, clinical profiles, pathway profiles, and image information. Computer-aided methods adequately capture the important features that are key to developing robust models (18). Most of the available methods possessed an obvious dependence on labeled data. However, in the clinical practice field, such training labeled dataset collection for these patients has been proven to be difficult, time-consuming, and often frustrating. The development of a precision medicine paradigm for these cancers is hampered by difficulty in obtaining tumor-related labeled data.

Indeed, labeled data inadequacy is a common problem for both precision medicine and clinical trial. Developing a novel method that can predict the therapeutic response and identify the biomarkers based on a minimal amount of sample is a task of top priority. Semi-supervised learning has proven to be a powerful paradigm for leveraging unlabeled data to mitigate the reliance on large labeled datasets. It is necessary to fully exploit the available class label information, especially in cases where few labeled data are available. Currently, semi-supervised learning is widely used in image identification. Songpa et al. proposed a simple and efficient method of semi-supervised learning for deep neural networks: Pseudo-Label (19). Timo et al. developed a self-assembling semi-supervised method under a variety of regularization and input enhancement conditions (20). Colin et al. proposed the semi-supervised framework MixMatch (21), which guessed low-entropy labels for data-augmented unlabeled examples and mixed labeled and unlabeled data using the MixUp method (22). All of these methods worked well in the field of image recognition, and data augmentation is an important factor in semi-supervised success. Data augmentation is one of the most skilled in deep learning, for example, translation and rotation (23) (24). The main purpose of data augmentation focused on increasing the sample size and generating a more diverse dataset (25). In principle, labels predicted by the model should be consistent for the samples generated by the same data augmentation, which is called consistency regularization (26) (27). In addition, semi-supervised learning is based on the consensus that the classification boundary of the classifier should not pass through the high-density region of the marginal distribution. That is forcing the classifier to predict with low entropy, which is also named entropy minimization (28). Inspired by these methods, we distill the advantages of successful available image identification algorithms and develop a semi-supervised method that is applied in the medical molecular field.

**Figure 1:**
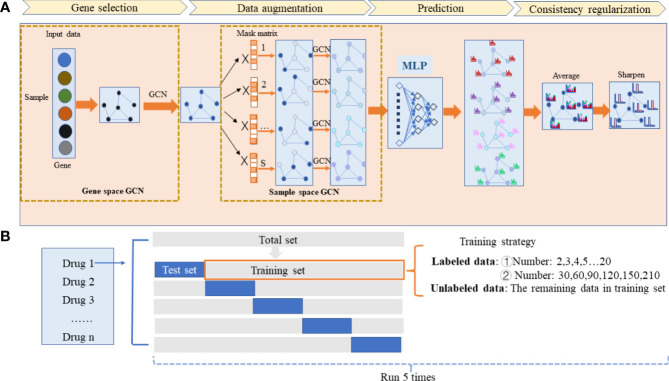
The workflow of the HiRAND framework and the analysis strategy. **(A)** HiRAND consisted of four main parts, including gene selection, data augmentation, prediction, and consistency regularization. Firstly, the feature embedding of gene expression profiles was obtained through GCN. Secondly, the multiple graph augmentations were generated by random propagation. Thirdly, the label prediction distribution was obtained through MLP. Fourthly, the final label results were used through consistency regularization. **(B)** For each drug response prediction task, we conducted 5-fold cross-validation to benchmark the predictive power of HiRAND. For the labeled data sample size setting, we considered two aspects: ① very few labeled sample size (2,3,4,5,6…20) and ② relatively large labeled sample size (30,60,90,120,150,210). The remaining data in the training set were treated as unlabeled data. This process was repeated five times to show the robustness of HiRAND.

We introduce Hierarchical Graph Random Neural Networks (HiRAND), a graph convolutional network (GCN)-based deep learning semi-supervised model, to predict drug response using transcriptomic data. HiRAND completes the information integration of the gene graph and the sample graph by GCN in order. In the gene spatial, the neighbor information is aggregated to generate new convolutional features based on the gene weight matrix and gene adjacency matrix. In the sample spatial, by dropping out a certain proportion of sample expression data several times, the sample’s information can pass to its neighbor along the pre-built sample adjacency matrix and generate a novel perturbation matrix based on GCN. The perturbation matrix is the augmentation version of the original expression data. By doing so, we achieve data augmentation for molecular expression data and improve the model generalization. An overview of HiRAND is illustrated in **Figure 1A**. In our results, HiRAND significantly outperformed classification models typically in most of the experiments, especially in the case of few labeled samples (the labeled sample size range is from 2 to 20). In addition, we provided interpretations of the genes that are selected by HiRAND to understand their drug-specific response mechanisms. Thus, HiRAND achieves superior predictive performance compared with established models and reveals the biological mechanism of drug response, with translational implications.

## Methods

### The overall structure of HiRAND

The proposed method HiRAND mainly consisted of four parts, including gene selection, data augmentation, multilayer perception (MLP) for prediction, and consistency regularization (**Figure 1A**). The initial input data of the model were the gene expression matrix *X*_input_ in a gene sample [sample means the nodes of the sample similarity graph *G* ^(^ *^n^* ^)^ throughout the text] format and the gene adjacency matrix *A^g^*. For clarity, we simply described the procedure of integrating the information using GCN in the graph data as shown in Equation 1. Consider a graph G=(V,E), where Vdenoted the vertices and E denoted the edges. The aggregation process of GCN was defined as:


(1) H=σ(X(A⊙W))

where *X* was input data denoting the characteristic matrix of the vertices, and *A* was the adjacent matrix denoting the relationship between nodes. *W* was layer-specific trainable weight matrix, and *σ* represented the activation function. Two types of graphs were used in HiRAND: gene interaction graph *G* ^(^ *^g^* ^)^ and sample similarity graph *G* ^(^ *^n^* ^)^, and convolution of those two graphs performed the gene selection and data augmentation, respectively. The consistency regularization was used to optimize the prediction consistency among *S* augmentation data. The pseudocode of HiRAND was provided in **Algorithm 1**.

Algorithm 1
**Input:**
*Adj_gene_
*= g × g, adjacent matrix for g genes
*Adj_sample_
* = *n* × *n*, adjacent matrix for *n samples X = n* × g, feature matrix for n samples and g genes
*S*: time of augmentation
*δ* = DropNote/dropout probability
*η =* learning rate
*f_mlp_
*: the model
**Function** HiRAND (*Adj_gene_
*, *Adj_sample_
*, *X, S, δ, η*):
 SetVariablesNull (*Z, ImportanceScore*)
 // *augment the data*
 **for** *s* in 1 : *S* **do**
   // sparse the input
*X*1 = SparseGCN    (X*V_weight_
*Adj_gene_
)
   // *perturb the data X*2 ~ DropNote (*X*1, *δ*)
   // *propagate the data*

$X3=\frac{1}{1\;+k}\sum_{k=0}^{K}Adj_{sample}^{k}X2$

   // *predict the label Z^s^
* = *f_mlp_
*(*X*3) **end**
 
$Z=f_{mlp}\left(\frac{1}{1\;+k}\sum_{k=0}^{K}Adj_{sample}^{k}X\right)$

 // *loss function L_total_
* = *L_label_
* + *L_unlabel_
*
 // *Importance score of genes*
 *ImportanceScore =* sum (abs(*V_weight_
* * *Adj_gene_
*))
 **return** *Z*, *ImportanceScore*
**Output:**
*Z:* the label distribution prediction
*ImprotenceScore:* the importance score of genes

### Gene selection

The main task of this layer was to aggregate the gene information along the gene adjacency matrix and achieve the gene selection. As Equation 2 showed:


(2) $$H^{\left(1\right)}=\sigma \left(X_{\text{input }}\left(A^{g}⊙W^{\left(0\right)}\right)\right)$$

*X*_input_ was n*g matrix, where each row represented a sample and each column denoted a gene. The gene adjacency matrix *A^g^* was g*g one-hot matrix, where $A_{ij}^{\left(g\right)}=1$ represented the interaction between gene *i* and gene *j* could be found in the HINT database, which was a database of high-quality protein–protein interactomes for humans (20). *σ*(·) denoted the Tanh activation function here. *W* ^(0)^ was a g*g trainable matrix, which contributed to the gene selection by assigning a higher weight value $W_{ij}^{\left(0\right)}$ to more critical genes. To this end, we constructed the gene importance score as Equation 3:


(3) $$I_{j}=\frac{\sum_{i}\left(A_{ij}^{\left(g\right)}⊙\left|W_{ij}^{\left(0\right)}\right|\right)}{\sum_{i}\sum_{j}\left(A_{ij}^{\left(g\right)}⊙\left|W_{ij}^{\left(0\right)}\right|\right)}$$

$A_{ij}^{\left(g\right)}$ and $W_{ij}^{\left(0\right)}$ were the elements of the *A^g^* and *W* ^(0)^ separately.

This output *H* ^(1)^ of the gene selection layer was still n*g matrix.

### Data augmentation

The purpose of this process was to complete data augmentation for the output *H* ^(1)^ matrix by performing the random propagation strategy. The sample adjacency matrix *A^n^* indicating the relationship of samples was used to store information about the sample graph structure. For constructing the *A^n^*, the similarity between sample *p* and sample *q* as evaluated by Equation 4:


(4) $${\mathrm{Sim}}_{pq}=\exp\left(-\frac{\rho^{2}\left(x_{p},x_{q}\right)}{\mu \epsilon_{pq}}\right)$$

where *x_p_* and *x_q_* denoted the g-dimensional vectors of gene expression profiles of sample *p* and sample *q*, respectively. *ρ*(·,·) was a function of calculating the distance between two different samples. *ρ*(·,·) was the cosine distance here. *µ* was the hyperparameter, which was set to 0.5 in this case. *ϵ_pq_* is a scale parameter and computed through Equation 5:


(5) $$\epsilon_{pq}=\frac{\mathrm{mean}\left(\rho \left(x_{p},N_{p}\right)\right)+\mathrm{mean}\left(\rho \left(x_{q},N_{q}\right)\right)+\rho \left(x_{p},x_{q}\right)}{3}$$

*N_p_* represented the neighbor’s expression vector of sample *p*, and similarly *N_q_* denoted the neighbor’s expression vector of sample *q*. Then, neighbors of a given sample were defined as the top 10 most similar samples based on the Sim*_pq_* according to the mild assumption that local similarities are more reliable than remote ones (29). The non-zero part in sample adjacency matrix *A^n^* of dimension n*n was constructed according to the Sim*_pq_*, and the diagonal elements are set to self-to-self similarity. Therefore, each sample in the *A^n^* was only connected with 10 neighbors at a certain distance.

Next, we randomly generated the binary mask *ϵ*_*p*_~ Bernoulli (1−*δ*) for each sample *p*, where *δ* was the drop rate. To effectively augment graph data, each sample’s expression data can be randomly dropped (referred to as Dropout) (30) by multiplying the samples’ expression vector with its corresponding mask: ${\tilde{x}}_{p}=\epsilon_{p}\cdot x_{p}^{\prime }$ , where $x_{p}^{\prime }$ denoted the feature matrix of sample *p* in *H* ^(1)^. Then, we adopt the GCN to propagate the neighbors’ information to each sample along the sample adjacency matrix *A^n^* as Equation 6:


(6) $$H^{\left(2\right)}=\sigma \left(\tilde{X}\left(A^{n}⊙W^{\left(1\right)}\right)\right)$$

where $\tilde{X}$ was the set of ${\tilde{x}}_{p}$ , and output matrix *H* ^(2)^ was a sample augmentation data. *A^n^* was the sample adjacent matrix, and *W* ^(1)^ was the weight matrix. As shown in **Figure 1A**, HiRAND yielded *S* sample data augmentations based on the *S* mask matrix, and here, *S* was set to 4.

It is well known that GCN only makes the convolution for the nodes that were directly connected in the graph data. In order to explore the role of the *m*-hop neighbor, we consider using the ${\bar{A}}^{n}$ instead of *A^n^* to receive the information of both near and distant neighbors in the sample graph data (Equation 7):


(7) $${\bar{A}}^{n}=\sum_{m=0}^{m}\frac{1}{m+1}A_{m}^{n}$$

$A_{m}^{n}$ was the adjacent matrix of the *m*-hop neighbor. ${\bar{A}}^{n}$ is the average of the power series of *A^n^* from order 0 to order *m*.

### MLP for prediction

After performing the data augmentation *S* times, we obtained S augmented data matrices $H_{s}^{\left(2\right)}$ (1≤ *s<* S). Then, these *S* augmentation datasets were fed into the MLP classifier separately as Equation 8:


(8) $${\tilde{Z}}^{\left(s\right)}=f_{mlp}\left(H_{s}^{\left(2\right)},\Theta \right)$$

Where Θ denoted the model parameters. MLP consisted of three layers: the input layer with *g* neurons (*g* means the number of columns of the input data), the hidden layer with 128 neurons, and the output layer with *C*neurons (*C*was the number of classes and was set to 2 here). For each sample, the probability of being assigned to each class denoted by ${\tilde{Z}}^{\left(s\right)}\in {\left[0,1\right]}^{n\times C}$ could be obtained.

### Consistency regularization

Having generated *S* prediction probability matrices from the previous layer, we proposed to optimize the prediction consistency among *S* outcomes. We first calculated the label distribution center ${\bar{Z}}_{p}$ by taking the average of all distributions (Equation 9):


(9) $${\bar{Z}}_{p}=\frac{1}{S}\sum_{s=1}^{S}{\tilde{Z}}_{p}^{\left(s\right)}$$

${\tilde{Z}}_{p}^{\left(s\right)}$ was the prediction result distribution of the sample *p* in the *s* data augmentation.

Next, inspired by the sharpening algorithm (21), the sharpening function (Equation 10) was utilized to reduce the entropy of the average label distributions. Specifically, the guessed probability ${\bar{Z}}_{pw}^{\prime }$ of sample *p* in the *w*th class is calculated by:


(10) $${\bar{Z}}_{pw}^{\prime }={\bar{Z}}_{pw}^{-\frac{1}{T}}/\sum_{c=0}^{C-1}{\bar{Z}}_{pc}^{-\frac{1}{T}},(0\leq w\leq C-1)$$

where *T*∈(0,1] , which aimed to control the sharpness of the categorical distribution. Therefore, the label probability approached one-hot encode label and was treated as the artificial label.

### Loss function

Our final loss function to be minimized consisted of two parts (Equation 11), and *λ* was the parameter that balanced the *ℒ*_lab _ and *ℒ*_unlab _ :


(11) $$ℒ={ℒ}_{\text{lab }}+\lambda {ℒ}_{\text{unlab }}$$

*ℒ*_lab _ was a cross-entropy term representing the classification error (Equation 12):


(12) $${ℒ}_{lab}=-\frac{1}{S}\sum_{s=1}^{S}\sum_{i=0}^{q-1}Y_{i}^{⊤}\log\;{\tilde{Z}}_{i}^{\left(s\right)}$$

where *q* denoted labeled nodes among *n* nodes, and *Y* was the true label of the labeled sample. *ℒ*_unlab _ was a penalty term for the regularization loss that denoted the distance between the artificial label and the predicted label averaged over all augmentations (Equation 13):


(13) $${ℒ}_{\text{unlab }}=\frac{1}{S}\sum_{s=1}^{S}\sum_{i=0}^{n-1}{\left\|{\bar{Z}}_{i}^{\prime }-{\tilde{Z}}_{i}^{\left(s\right)}\right\|}_{2}^{2}$$

${\bar{Z}}_{i}^{\prime }$ was the guessed probability, and ${\tilde{Z}}_{i}^{\left(s\right)}$ was the predicted probability for a given sample.

In the HiRAND training phase, the analysis strategy was shown in **Figure 1B**. For each drug response prediction, 80% of the whole samples were seen as the training set, including labeled data and unlabeled data, and we selected 20% of the total samples as the test set. We selected K samples of the training set as labeled samples to fine-tune the model and the remaining samples as unlabeled samples. In *K* value setting, we primarily considered two aspects. On one side is exploring the model performance in the case of few labeled data, and *K* is set to range 2–20. On the other side, we would like to see the performance comparison with the baseline model when more samples are given, so we set the labeled sample size as {30,60,90,120,150,180,210}. We conducted 5-fold cross-validation to evaluate the predictive power of HiRAND and ran this process five times to show the robustness. Full details of data preparation and the comparison of available methods are given in the **Supplementary Methods** subsection.

## Results

### The performance of HiRAND on the simulation data

To assess the performance of HiRAND, we simulated an expression matrix of 500 samples with 1,000 variables. Firstly, a protein–protein interaction (PPI) network-like adjacency matrix representing the connection strength among variables was constructed. Specifically, we employed the preferential attachment algorithm proposed by Albert (31) to generate a scale-free feature graph of 1,000 nodes. The distance matrix D recording pairwise distances among all nodes was then calculated, whose dimension was 1,000 * 1,000. Next, we derived the covariance matrix Σ*_ij_* by transforming the distances matrix D between nodes according to Equation 14:


(14) $$\Sigma_{ij}=0.7^{D_{ij}},i,j=1,\ldots ,1000$$

Based on the covariance matrix Σ*_ij_*, 500 multivariate Gaussian samples were obtained as input matrix **X**=(**x**_1_,…,**x**_500_)*^T^* followed by Equation 15:


(15) $$x_{i}\sim N(0,\Sigma ),i=1,\ldots 500$$

To generate the outcome variable, we defined 20 features as the true predictors randomly. A set of parameters *β*=(*β*_1_,…,*β*_20_)*^T^* and an intercept *β*_0_ were sampled in range (1, 1.5). We also made some of the parameters negative so that both positive and negative coefficients can be accommodated, which was more consistent with the situation in practical applications. Ultimately, the outcome variable y was calculated as in Equations 16 and 17:


(16) $$\mathrm{Pr}\left(y_{i}=1|x_{i}\right)=\eta^{-1}\left(x_{i}^{T}\beta +\beta_{0}\right)$$

(17) $$y_{i}=J\left(\mathrm{Pr}\left(y_{i}=1|x_{i}\right)>m\right),i=1,\ldots 1000$$

where *η* ^−1^(*x*)=(0.7*ϕ*(*tan h*(*x*))+0.3*ϕ*(*x* ^2^))*(1+*e* ^*x*^)^2^ , in which *η* ^-1^(*x*) was the link function and *m* was the median of *y*. Thus, we generated the outcome variable *y*, which was the binary variable.

**Figure 2:**
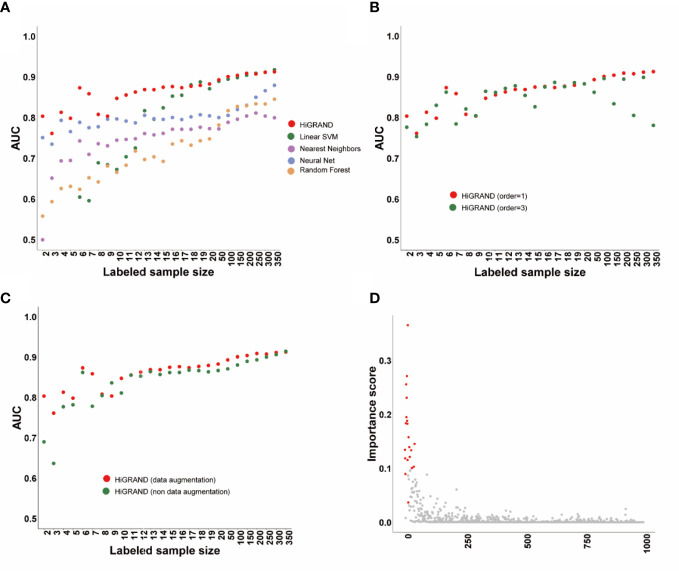
The performance of the HiRAND method on the simulation data. **(A)** The AUC of HiRAND and baseline models over all of the experiments on the simulation data. **(B)** The influence of graph convolution step size on the AUC. **(C)** The influence of data augmentation on the AUC. **(D)** The gene selection performance of HiRAND, where red represented differential variables and gray represented non-differential variables.

We applied HiRAND to the simulation data following the analysis strategy described before. We found that the area under the curve (AUC) of HiRAND is higher than all of that of compared methods in the presence of very few labeled samples, such as less than 20. The AUC of the compared methods has been improving rapidly by adding the labeled sample, especially in SVM, which roughly gets the same AUC with HiRAND trained with more than 20 labeled samples (**Figure 2A**). As can be seen, the HiRAND algorithm performed well with very few labeled data. In **Figure 2B**, we showed the effect of the step size in sample graph convolutions and, consequently, on the prediction performance. Outside of a few instability results, the model with 1 step size is better than with 3 step sizes. Moreover, to further explore the effects of data augmentation strategy on prediction performance, we deleted the data augmentation layer from HiRAND as an ablation experiment. As **Figure 2C** showed, data augmentation indeed improved the predictive power of the model. **Figure 2D** demonstrated the capacity for feature selection of HiRAND; red represented the differential features, and gray represented the non-differential features. The importance scores of the differential features were significantly higher than those of the non-differential features.

### HiRAND provided a competitive performance in drug response prediction

We further evaluated the HiRAND classification performance on the drug response data. After merging the cell line expression data and pharmacological response, the number of cell lines for each compound was shown in **Figure S1**.

According to the number of cell lines, 62 compounds with more than 600 cell lines were picked, whose molecular targets focus on 20 pathways. For each individual drug, we trained the model with the different labeled samples in accordance with the setup that was mentioned, and there were a total 1,612 experiments for all 62 drugs. The prediction ability was measured by AUC, accuracy, and F1 score. We compared our model with four classic methods, including neural net, nearest neighbor, SVM, and random forest. The overall result of the comparison between HiRAND and other methods was shown in **Table 1**. For an experiment, the result was labeled “Win” if the AUC of HiRAND was more than that of the control method and the difference was statistically significant. Similarly, the result was labeled “Tie” if the difference was not statistically significant, and it was labeled “Loss” if HiRAND performed worse than the control number. With these three measures, the HiRAND model won most of the pairwise comparisons (**Table 1**).

**Table 1:** Performance comparison of HiRAND and competitive methods.

	AUC				F1				Accuracy			
	Neural Net	Nearest Neighbor	SVM	Random Forest	Neural Net	Nearest Neighbor	SVM	Random Forest	Neural Net	Nearest Neighbor	SVM	Random Forest
Win^a^	833	1196	1222	1220	1182	1570	1286	1294	655	1263	1193	1197
Loss^b^	1	0	2	4	0	0	11	3	2	3	9	5
Tie^c^	778	416	388	388	430	42	315	315	955	346	410	410

a Win represented that the performance of HiRAND was better than that of the competitive method.

b Loss represented that the performance of HiRAND was worse than that of the competitive method.

c Tie represented that the performance of HiRAND was equal to that of the competitive method.

**Figure 3:**
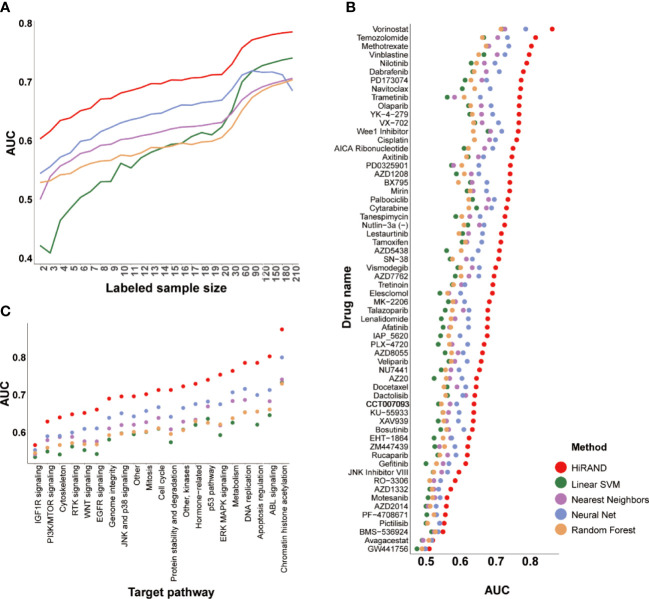
The predictive performance of HiRAND on drug response data. **(A)** The plot showed the average model AUC across all of the drug response models trained by different labeled samples. We considered the number of labeled samples from 2 to 20 as the first test level and from 30 to 210 as the second test level. The AUC of HiRAND was displayed separately for each drug **(B)** and target pathway **(C)**.

In the five 5-fold cross-validation experiments, HiRAND achieved satisfactory performance; the average AUC keeps improving as labeled samples were added, especially in cases of very few labeled data (**Figure 3A**). In the meantime, the prediction AUC of HiRAND is consistently higher than that of compared methods. Furthermore, we demonstrated the prediction presentation of models on each compound separately. As seen from **Figure 3B**, for some compounds like GW441758, no method can appear workable to complete the classification, which suggests that the gene expression information is insufficient for predicting the response of this compound. Notably, we found that some drugs involving vorinostat and temozolomide showed much higher AUC (>0.8) compared to other compounds, suggesting that the gene expression data can be used for pretreatment screening of patient candidates to these drugs. As expected, the AUC of HiRAND was consistently higher than the baseline models throughout the 62 drug predictions. Furthermore, we grouped the compounds by therapeutic target based on the drug-target profiles and explored the drug response predictable using the gene expression data (**Figure 3C**). We found that the response of IGFR pathway-related drugs showed the worst performance, including the BMS-536924 compound. Conversely, the histone deacetylase (HDAC)-related drugs, such as vorinostat, obtained the highest AUC. For demonstrating the AUC comparison between HiRAND and four compared methods in the individual drug in more detail, we plotted the heatmap, where red, white, and blue represented HiRAND win, tie, and lose separately. As **Figure S2** showed, HiRAND maintained its competitive advantage in most cases. So did results in accuracy (**Figure S3**) and F1 score (**Figure S4**), which further confirmed the discriminatory capacity of our method.

### Ablation experiments on drug response prediction

We selected the best AUC performance for the top five drugs, including vorinostat, temozolomide, methotrexate, vinblastine, and nilotinib, to conduct ablation studies on HiRAND to investigate the contribution of different parts of our model. The information of the cell line that engaged in model building for the top drugs was summarized as **Figure S5**.

**Figure 4:**
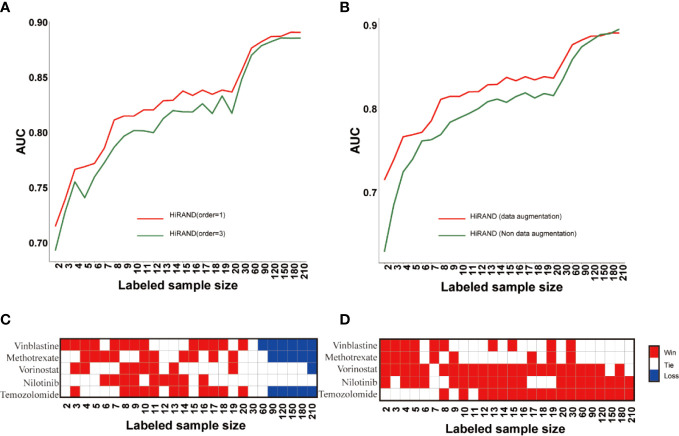
The ablation studies for examining the contributions of different components in HiRAND. **(A)** The AUC comparison of different convolution step lengths; red line and blue line represented the one step and three steps, respectively. **(B)** The AUC comparison of data augmentation; red line and green line represented the training with and without data augmentation. The contrast results of HiRAND and **(C)** HiGCN and **(D)** AffinityNet across five drugs. Red, white, and blue represented HiRAND win, tie, and loss in the comparison, respectively.

Firstly, to explore the role of step size in sample graph convolution, we designed two experimental studies on different step sizes. Consistent with the simulation experiment result, HiRAND with 1 step size was markedly more accurate than HiRAND with 3 step sizes, especially with a small number of labels (**Figure 4A**). For sample graph convolution, the effect of first-order neighbors on a special node is enough; inclusion of multi-order neighbor information may increase the risk of information bias, which hampered the sample classification. In addition, to further explore the role of data augmentation strategy in classification, we completed an ablation experiment in which we deleted the data augmentation layer from HiRAND. Although the advantage of data augmentation disappeared when the labeled sample size was more than 120, we observed significant improvements during training on very few labeled data (**Figure 4B**). The data augmentation was carried out depending on the sample graph convolution (propagation) and mask matrix and integrating information from neighbors. During the data augmentation process, the bias can be instilled into model construction inevitably. As we all know, data augmentation promoted the diversification of the input data to some extent and optimized the classification performance. During training with very few labeled samples, the bias is tolerable because of alleviating the dilemma in limited labeled samples. However, the information carried by amounts of labeled data is enough to distinguish distinct classes when more labeled samples were involved in model building; the data augmentation highlights the disadvantage of the bias. To investigate the effects of activation functions on prediction performance, we trained the model with Relu and Tanh (default setting) functions separately. The results also indicated that different activation functions had little effect on the result of HiRAND (**Figure S6A**). To explore the function of various numbers of MLP hidden layers, we compared the performance of default setting HiRAND [128, 32] to another MLP layer setting, including MLP hidden units [64, 32] and MLP hidden units [128,64,32]. As **Figure S6B** showed, all comparisons ended in a tie. In addition, 129 comparisons ended in a tie, and one comparison ended in a win (**Figure S6C**). Therefore, we speculated that different MLP layer settings might have little influence on the performance of HiRAND.

Then, we compared our model with other semi-supervised methods, including AffinityNet (32) and HiGCN (33). The average prediction AUC over the 5-fold is used as the final performance measure. As **Figures 4C, D** showed, HiRAND achieved higher AUC than all other methods, especially when the amount of labeled data is limited. Clearly, as the number of labeled data increased, the AUC gap between HiRAND and contrast semi-supervised method gradually narrowed. We noted that AffinityNet and HiGCN could achieve remarkable AUC performance using large quantities of labeled data, even exceeding the AUC of HiRAND. Once again, our results verified that the data augmentation strategy does not entail a direct prediction advantage when a lot of labeled samples were treated as the training set because the disadvantage of introducing noise into the model covered the advantage of enhancing the data diversity.

### HiRAND selected the important genes for predicting drug response

**Figure 5:**
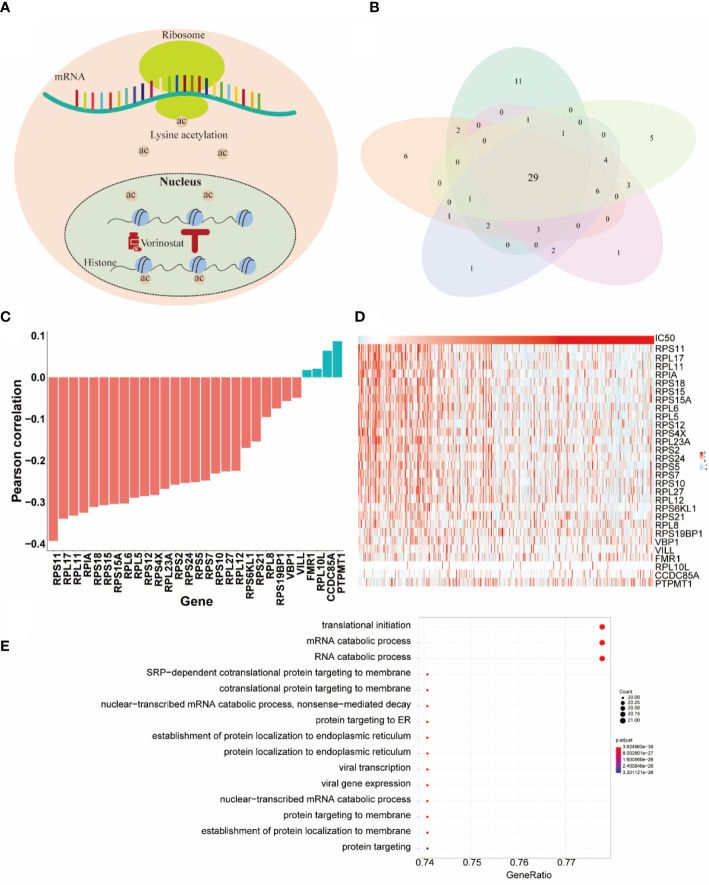
The HiRAND interpretation to identify predictive markers. **(A)** Schematic representation of acetylation sites. **(B)** The intersections of genes across five repeat experiments were treated as the predictive markers. **(C)** The Pearson correlation between the 29 predictive markers and IC50 value. **(D)** The heatmap of the gene expression of the 29 predictive markers. **(E)** GO analysis results of the 29 predictive markers.

In this section, we paid more attention to the role of selected genes by HiRAND. We investigated the important genes selected depending on the vorinostat response prediction, which had the best predicted results. Vorinostat, a histone deacetylase inhibitor (HDI), promotes cell cycle arrest (34). In the past decade, proteomic analyses have revealed that non-histone proteins are frequently acetylated and constitute a major portion of the acetylome in mammalian cells (**Figure 5A**). Indeed, non-histone protein acetylation is involved in key cellular processes relevant to physiology and disease, such as gene transcription, DNA damage repair, cell division, signal transduction, protein folding, autophagy, and metabolism (35). Xu et al. (36) found that acetylation of ribosomal proteins likely improved their stability and possibly translational efficiency of ribosomes. We completed five repeated experiments trained by 20 labeled samples to select the top 50 genes based on important scores. As **Figure 5B** showed, 29 genes appeared in all five experiments and were defined as the key genes for vorinostat. The correlation analysis for the 29 key genes with IC50 value was shown in **Figure 5C**. We found that 23/29 genes belong to the ribosomal protein (RP) gene family, in which 22 RP genes were negatively correlated with IC50 value, and the strongest correlation value was -0.39. These results validated our hypothesis that ribosomal protein was acetylated under the influence of vorinostat to accelerate translation efficiency, which exerts a beneficial effect on survival. The overexpression of most genes in the lower IC50 value (**Figure 5D**) is consistent with our hypothesis. Moreover, we plotted the top 15 pathways depending on the Gene Ontology (GO) analysis (**Figure 5E**) of the 29 key genes, and most pathways were involved in translation and protein anchoring. These results reaffirmed our hypothesis and showed the gene selection capability of HiRAND. In addition, the relationship between vorinostat and ribosome was also confirmed in the published literature. The GO analysis showed that the active probe-binding proteins were highly enriched in the translation elongation and ribosome-associated pathways, and the probe was designed by connecting HDIs (such as vorinostat) (37). Marks (38) reported that the vorinostat-related acetylation sites were identified on proteins that regulate ribosome formation and function. Houston et al. (39) demonstrated that acetyl-CoA depletion alters the integrity of the nucleolus, impairing ribosomal RNA synthesis. This nucleolar remodeling appears to be mediated through the HDACs (39). Overall, these further validate our result of the 29 key genes.

## Discussion

Ranging from drug research and development (R&D) to precision medicine, obtaining the prognosis and treatment response labels of patients is often difficult and expensive. In this paper, we developed HiRAND, the GCN-based deep learning method for drug response prediction that enabled semi-supervised classification and feature selection using very few labeled data. One of the highlights of HiRAND is aggregating the information from both gene graph spatial and sample graph spatial simultaneously. Another major contribution is achieving data augmentation in the expression profile and overcoming the dilemma of the limited labeled sample size. Overall, our method performs significantly better than other competitive methods on the 5-fold cross-validation experiments, whether in multiple drug response predictions and translation scenarios. Notably, the advantage of HiRAND becomes more pronounced, especially in models trained using few labeled samples.

This paper provided a new idea for the exploration of predicting drug response using very few labeled data. Along this line of thinking, future research could focus on designing updated model versions to speed up the clinical application process (i.e., transferring the cell line to patient level). Ideas like the HiRAND method might be expected to work well in the clinical scenario. Traditional randomized drug-centered clinical trials are cumbersome, costly, and require large numbers of patients to demonstrate a clinical benefit (40). Computer-aided methods could help obtain and analyze information from various data sources and generate statistically valid drug sensitivity prediction models at all stages of drug development (41). A semi-supervised method allows us to overcome the problem of a small labeled sample size in this process and provides constructive advice for next stage study. Thus, the clinical success rates could improve by optimizing targeted therapy selection, patient enrollment, and stratification (42, 43). Further clinical trials lie in moving from drug-centric to patient-centric individualized combination therapy. A second compelling application is in clinical contexts seeking to implement precision medicine for individual patients. The strategy of matching drugs to patients on the basis of molecular features should begin earlier in the disease progression (i.e., to identify the right drug for the right patient). Classic predictive models have been shown to have limited predictive power for these two cases because of an inability to obtain large samples of well-characterized clinical data, which are the expression profiles matched with the outcome label like drug response. Our approach is one way of overcoming the problem of small labeled sample sizes and low reliability inherent to previous precision medicine and drug R&D studies. We can select the more precise candidates for a certain treatment using HiRAND, and the cure rate will hopefully increase. On the other hand, the time frame from development to routine clinical may be shortened to enhance the pace of drug discovery and development using the HiRAND method.

At present, studies have shown that HDACs can influence a multitude of physiological pathways in different cells. HDACs regulate non-histone proteins [adhesion proteins (44), transcription factors (45), cellular proteins (46), DNA-repair proteins (47), and cell signaling and viral proteins (48)] according to their acetylation state. HDIs (such as vorinostat) selectively modulate gene transcription through the changes in the structure of proteins involved in the transcriptional machinery. Vorinostat is the first-generation HDAC pan-inhibitor belonging to the hydroxamic acid group of HDIs approved by the Food and Drug Administration (FDA) (49). It has been demonstrated that vorinostat showed the antiproliferative activity of human cancer cell lines, including lung cancer (50), ovarian cancer (51), breast cancer (52), skin cancer (53), and so on. In this paper, we screened 29 key genes related to vorinostat using our HiRAND. After performing enrichment analysis for 29 key genes, we speculated that vorinostat might exert its activity by acting on the acetylation of non-histone (ribosomal proteins). This hypothesis will require a future investigation.

From the results of the ablation experiments, the multiple graph data augmentation strategy alleviated the low-sample size problem to some extent, based on which we utilized consistency regularization to improve the model’s generalization on unlabeled data. As expected, data augmentation made the performance better compared to standard networks trained without data augmentation. Extensive data augmentation was performed to overcome the limitation of our training data (54). Data augmentation was critical to avoid overfitting and maximize the generalization accuracy to unseen data (55). Thus, data augmentation can achieve good results not only in image identification but also in molecular pattern recognition. Secondly, the weight matrix in the gene graph convolutional contributed to the gene selection and improved the prediction performance. Moreover, we also observed that drug responses that target some pathways were better predicted than others, such as the response of the chromatin histone acetylation-related drug was predicted well. The reason was input data were obtained with the mRNA expression profile in the present study, which was insufficient to predict drug response for some compounds. A worthy future direction would be to enrich the input data by applying the fusion method for knowledge integration from multi-omics data. Another direction would be to better understand the relationship between the predictability of a drug and pharmacological properties, including the regulatory mechanism of drug targets and signaling pathways. After refining the input data, HiRAND will potentially predict the response of more drugs precisely.

To sum up, we introduced the HiRAND semi-supervised deep learning framework that can serve as an application for drug response prediction using few labeled samples. Meanwhile, the model could be used in the future to stratify patients based on the learned classification features, providing an important analysis tool for future applications in precision oncology and beyond.

## Conclusion

We proposed a novel semi-supervised framework HiRAND to predict the responders of treatment in precision medicine. By integrating information from both feature graphs and sample graphs, HiRAND clearly outperformed existing methods and demonstrated its capability in personal treatment decision-making. Not only this, this model is helpful for discovering outcome-oriented biomarkers by incorporating a trainable weight matrix. In addition, we introduced a data augmentation strategy into molecular biology to augment the biodata and overcome the small labeled sample size limitation. Our future effort will focus on considering more omics data into model building and increasing the performance toward biomarker discovery.

## Data availability statement

The original contributions presented in the study are included in the article/**Supplementary Material**. Further inquiries can be directed to the corresponding authors.

## Author contributions

Conceptualization: YH and KL; Methodology: YH, ZR, LZ, ZX, JJ, JH, WL; Supervision: KL, YH; Writing: YH; Review & editing: all; Code writing: YH, ZR, and LZ; Validation: YH, ZR, LZ, ZX. All authors contributed to the article and approved the submitted version.
