# Supplementary material for: HiRAND: A novel GCN semi-supervised deep learning-based framework for classification and feature selection in drug research and development
Source: Front Oncol. 2023 Jan 26;13:1047556. doi: 10.3389/fonc.2023.1047556 (PMC9909422; doi:10.3389/fonc.2023.1047556)
Supplement: Supplementary file 1 [file DataSheet_1.pdf]

Supplement Files

Methods

1 Real data preparation

Our study integrated two public databases, including Genomics of Drug Sensitivity in Cancer (GDSC) and Cancer Cell Line Encyclopedia (CCLE) data. IC50 values were seen as the outcome variable to assess the relative sensitivity of each cell line to each drug and were provided from GDSC database. CCLE database provided gene expression profiles for 1019 cancer cell lines. Finally, we curated a dataset comprising 37680 instances a cross 626 cancer cell lines and 62 drugs (Figure S1). All relevant information used in this study were summarized in Supplementary Table S1. Each instance denoted a drug treatment of a cancer cell line. The HiRAND was constructed for predicting how the cell lines respond to the drug treatment under study. It is possible to divide drug responses into two categories: “sensitivity" and “non-sensitivity”. However, we discovered from the density plot of IC50 that drugs have different distribution (Figure S7). Drug response labeling decisions can probably be based on the data of the individual drug. For this reason, we used the quartile of the observed IC50 values as a data-driven threshold. We labeled an instance as “sensitivity” if its IC50 is smaller than the first quartile overall the cell lines for an individual drug. We labeled an instance “non-sensitivity” if its IC50 is equal to or larger than the third quartile overall the cell lines for an individual drug.

2 Model evaluation and analysis strategy

We used the Python package ‘scikit-learn’ (http://scikit-learn.org/stable/index.html) to

implement four conventional machine learning methods: random forests, conventional neural networks (that is multilayer perceptron (MLP), consisting of three layers: input layer, hidden layer and output layer), K nearest neighbors (KNN) and SVM linear regression as competing methods. The default parameters of each method were used for model comparison. In addition, we validated the performance of all models using standard 5-fold cross-validation (5-CV). For each training, data set is randomly divided into a training set or a test set, so we repeated this data partitioning 5 times and reported the average and standard deviation of prediction performance over all of these replicates. For classification experiments, the area under the receiver operating characteristic curve (AUC), Accuracy and F1 score were used to measure performance of HiRAND.

Code for implementation details used in the analysis are presented in https://github.com/Kaplan-semi/HiRAND.

Figures

Figure S1: The number of cell lines treated by individual drug.

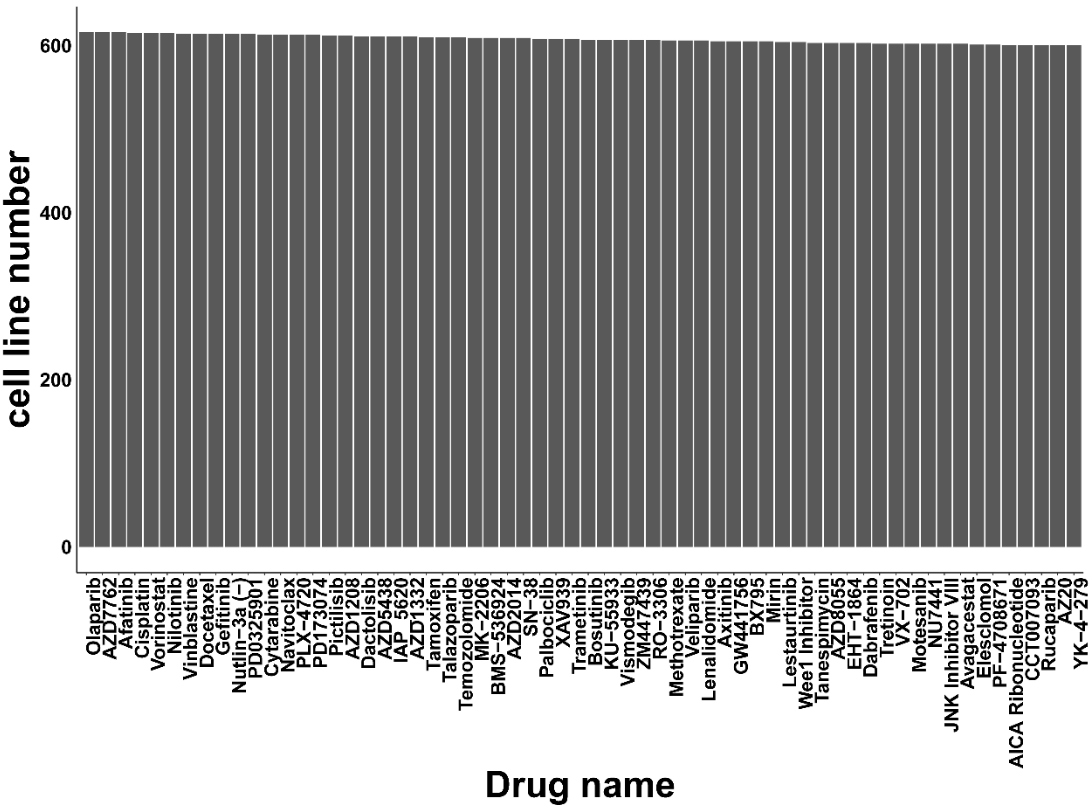

Figure S2: The AUC result comparison between HiRAND and other methods, which including (A) Neural Net, (B) Nearest Neighbor, (C) SVM and (A) Random Forest.

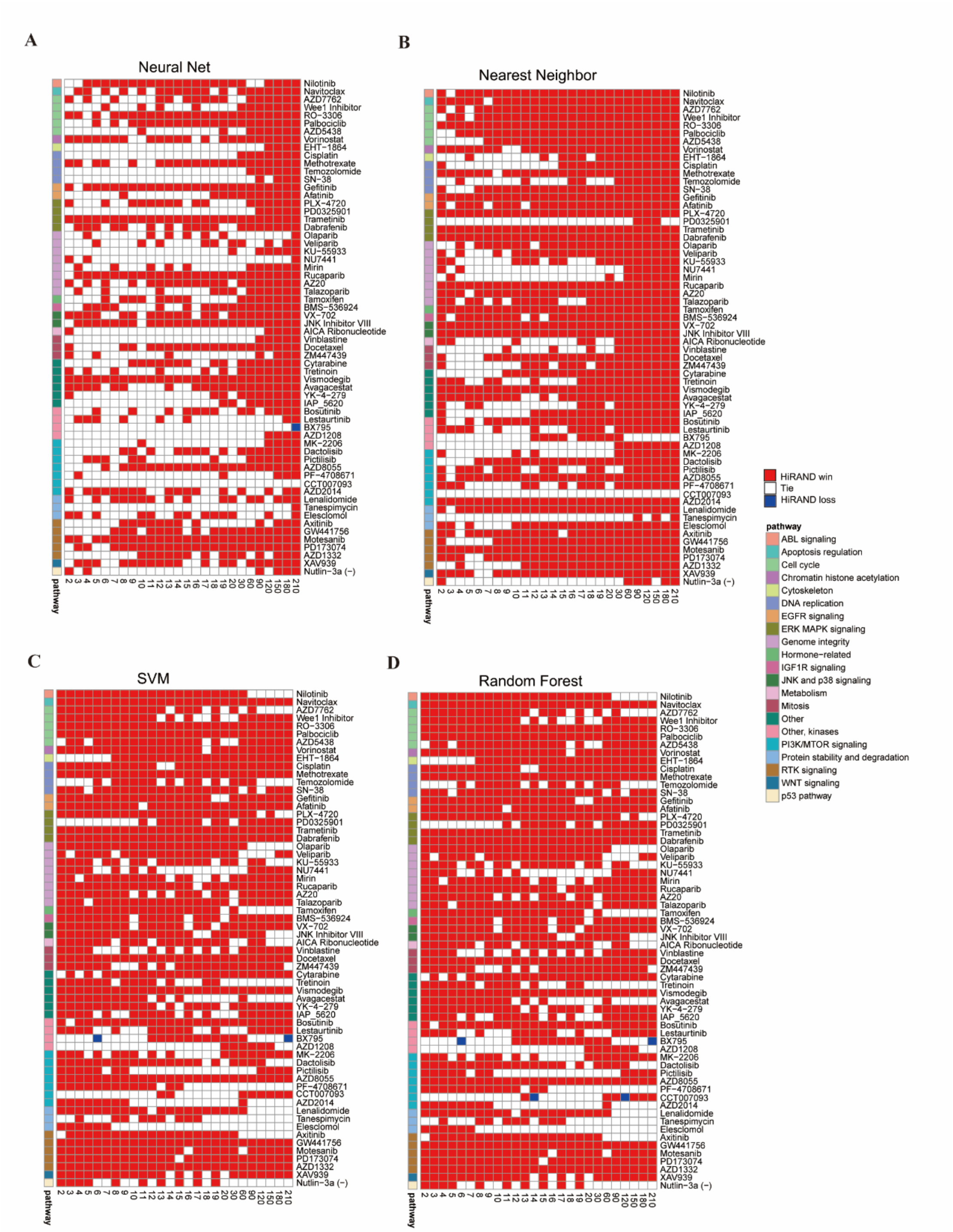

Figure S3: The predictive performance of HiRAND on drug response data. (A) The plot showed the average model Accuracy across all the drugs response model trained by different labeled samples. We considered the number of labeled samples from 2 to

20 as the first test level, and from 30 to 210 as the second test level. The Accuracy of HiRAND was displayed separately for each drug (B) and target pathway (C).

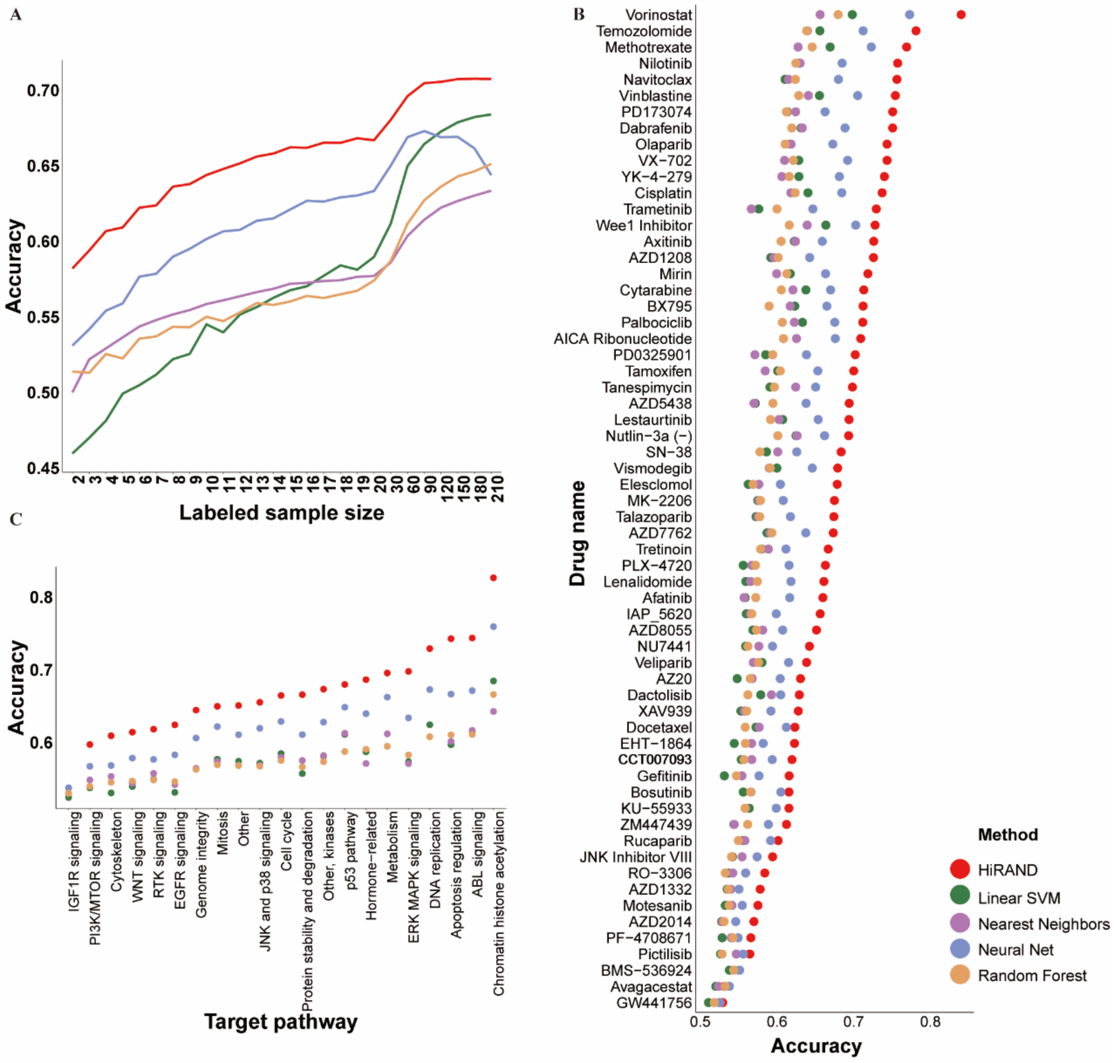

Figure S4: The predictive performance of HiRAND on drug response data. (A) The plot showed the average model F1 score across all the drugs response model trained by different labeled samples. We considered the number of labeled samples from 2 to 20 as the first test level, and from 30 to 210 as the second test level. The F1 score of HiRAND was displayed separately for each drug (B) and target pathway (C).

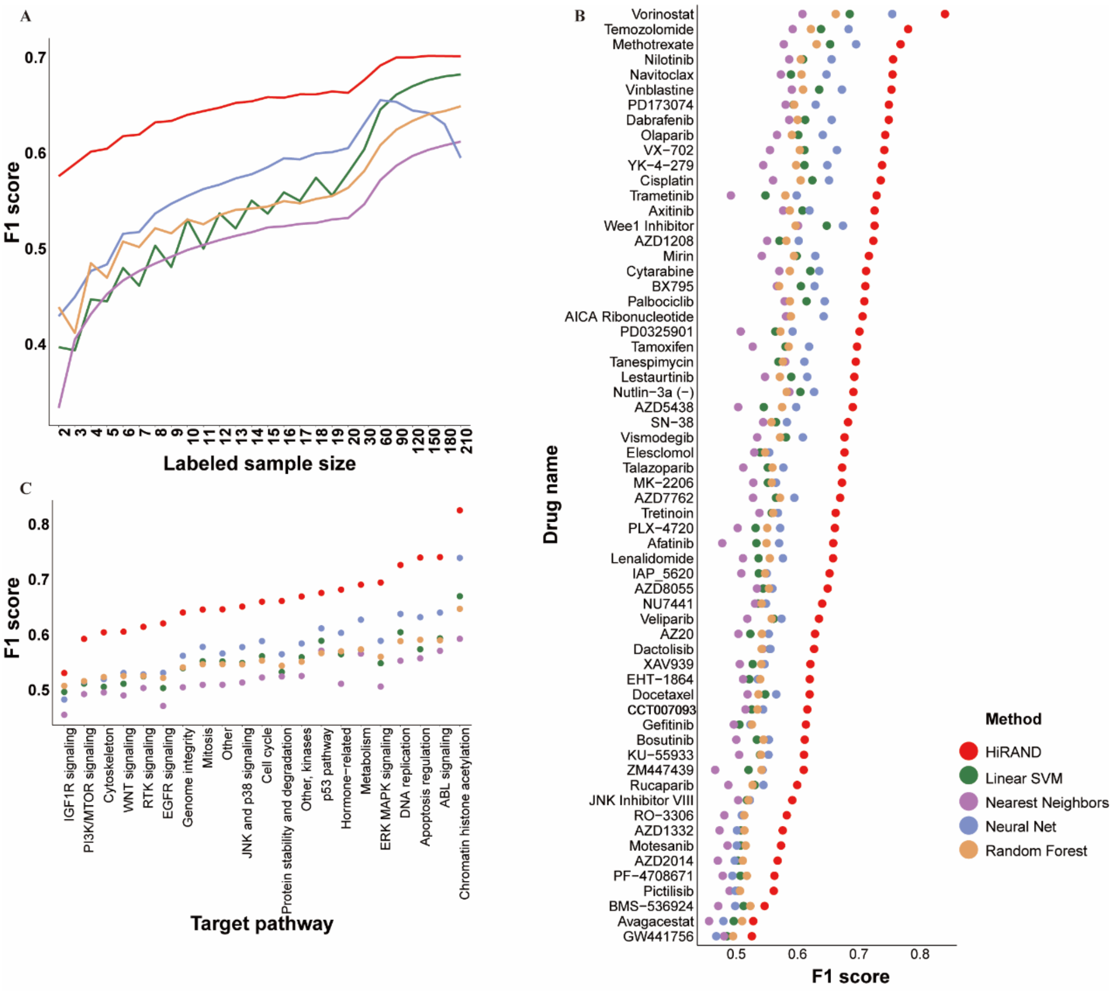

Figure S5: The information of cell line that engaged in model building for top 5 drugs: including Vinblastine, Methotrexate, Vorinostat, Nilotinib and Temozolomide. The abscissa represents the cell lines, and the ordinate represents the differential cancer types. Red represented cell line was used for model construction.

Vinblastine

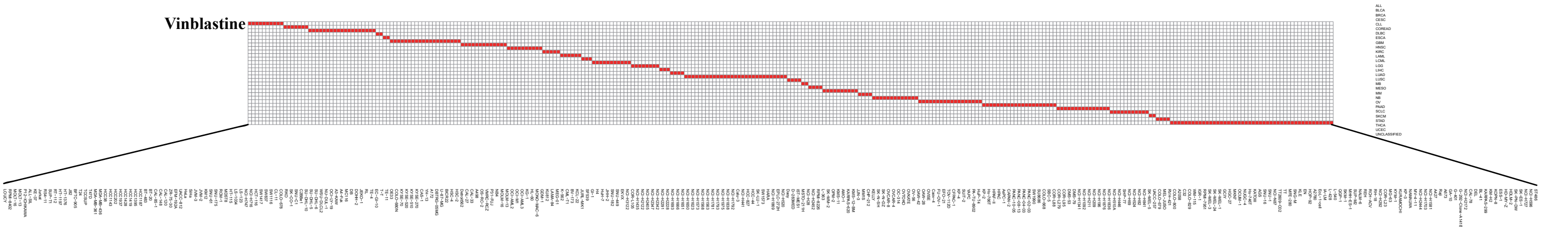

Methotrexate

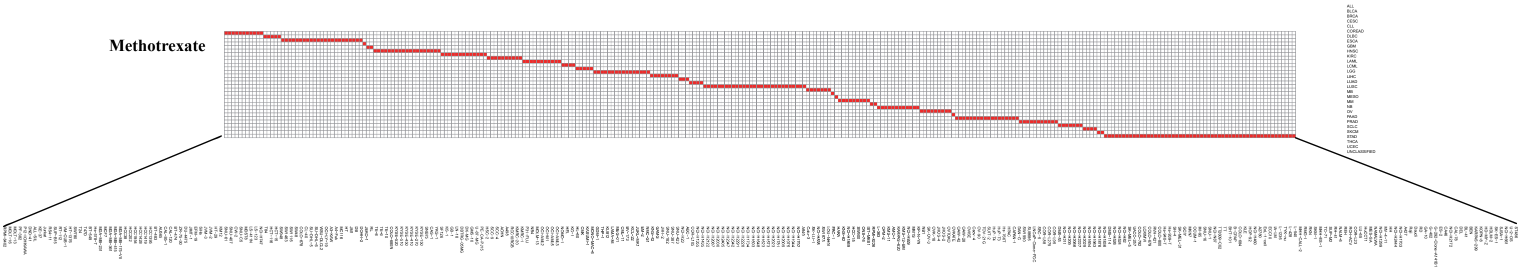

Vorinostat

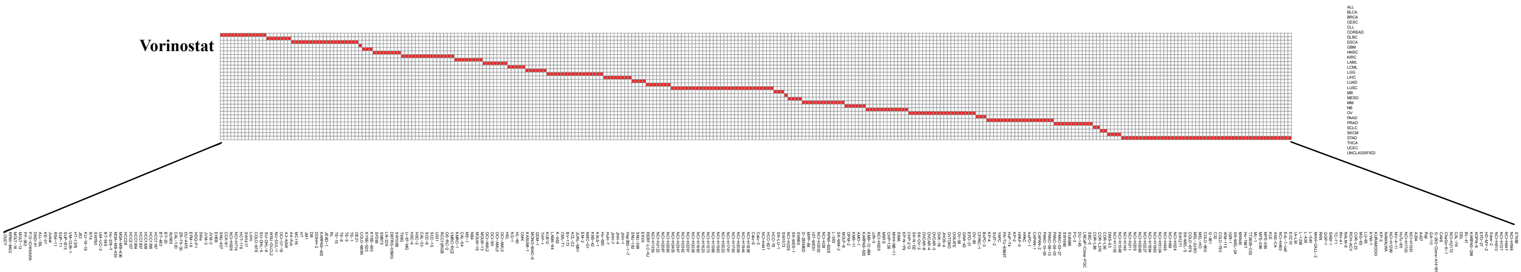

Nilotinib

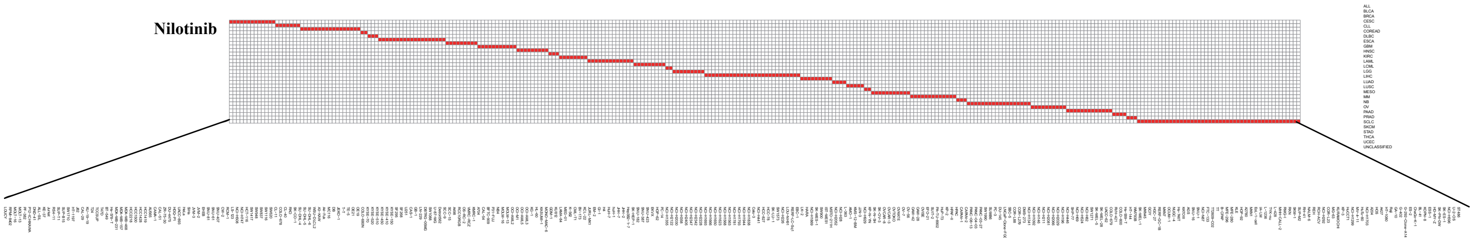

Temozolomide

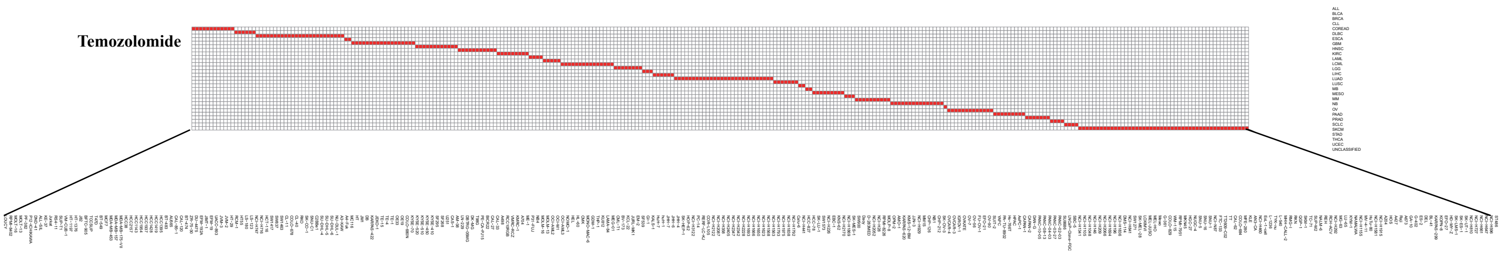

Figure S6: The AUC performance comparisons of the different activation function (A), different MLP layer settings (B, C) to the default setting of HiRAND.

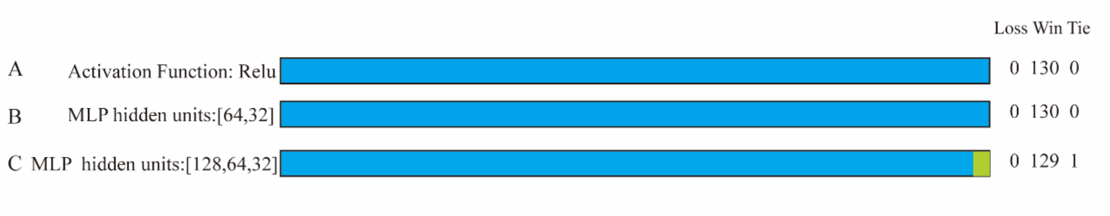

Figure S7: The density plot of IC50 value of individual signaling pathway related drugs.

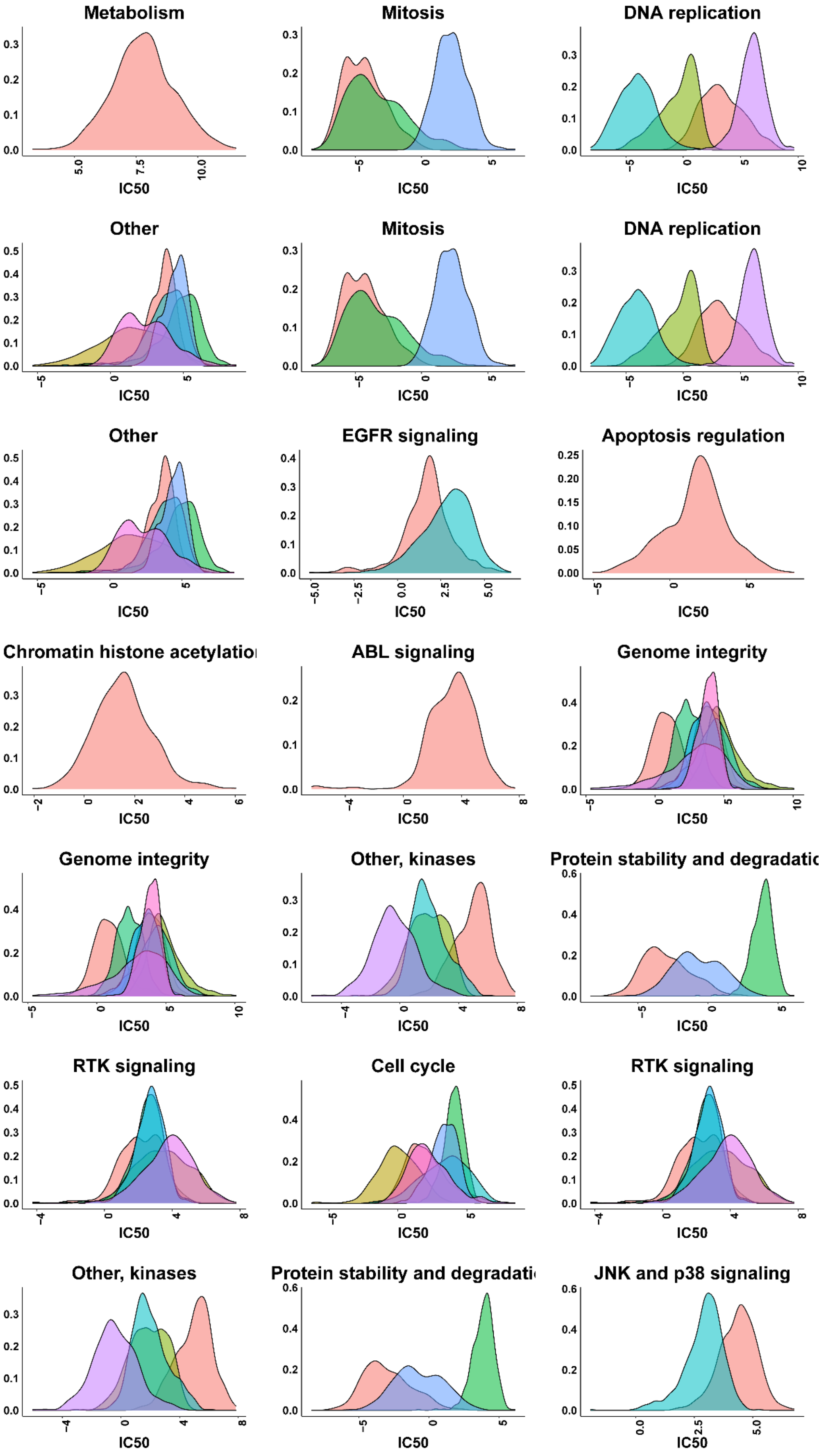

Metabolism
IC50
Mitosis
IC50
DNA replication
IC50
Other
IC50
Mitosis
IC50
DNA replication
IC50
Other
IC50
EGFR signaling
IC50
Apoptosis regulation
IC50
Chromatin histone acetylatio
IC50
ABL signaling
IC50
Genome integrity
IC50
Genome integrity
IC50
Other, kinases
IC50
Protein stability and degradatic
IC50
RTK signaling
IC50
Cell cycle
IC50
RTK signaling
IC50
Other, kinases
IC50
Protein stability and degradati
IC50
JNK and p38 signaling
IC50

Table S1: Detailed information about information of each drug obtained from the GDSC.
